# easySCF: a tool for enhancing interoperability between R and Python for efficient single-cell data analysis

**DOI:** 10.1093/bioinformatics/btae710

**Published:** 2024-11-25

**Authors:** Haoyun Zhang, Wentao Zhang, Shuai Zhao, Guangyu Xu, Yi Shen, Feng Jiang, An Qin, Lei Cui

**Affiliations:** School of Biomedical Engineering, Shanghai Jiao Tong University, Shanghai, 200240, China; School of Mathematical Sciences, Shanghai Jiao Tong University, Shanghai, 200240, China; Collaborative Innovation Centre of Regenerative Medicine and Medical BioResource Development and Application Co-constructed by the Province and Ministry, Guangxi Medical University, Nanning, 530021, China; Collaborative Innovation Centre of Regenerative Medicine and Medical BioResource Development and Application Co-constructed by the Province and Ministry, Guangxi Medical University, Nanning, 530021, China; Jiangsu Key Laboratory of Marine Pharmaceutical Compound Screening, College of Pharmacy, Jiangsu Ocean University, Lianyungang 222005, China; Shanghai Research and Development Center, UxBioInfo, Shanghai, 201100, China; Shanghai Key Laboratory of Orthopaedic Implants, Department of Orthopaedics, Shanghai Ninth People’s Hospital, Shanghai Jiao Tong University School of Medicine, Shanghai, 200125, China; Collaborative Innovation Centre of Regenerative Medicine and Medical BioResource Development and Application Co-constructed by the Province and Ministry, Guangxi Medical University, Nanning, 530021, China; Shanghai Key Laboratory of Orthopaedic Implants, Department of Orthopaedics, Shanghai Ninth People’s Hospital, Shanghai Jiao Tong University School of Medicine, Shanghai, 200125, China

## Abstract

**Summary:**

This study introduces easySCF, a tool designed to enhance the interoperability of single-cell data between the two major bioinformatics platforms, R and Python. By supporting seamless data exchange, easySCF improves the efficiency and accuracy of single-cell data analysis.

**Availability and implementation:**

easySCF utilizes a unified data format (.h5 format) to facilitate data transfer between R and Python platforms. The tool has been evaluated for data processing speed, memory efficiency, and disk usage, as well as its capability to handle large-scale single-cell datasets. easySCF is available as an open-source package, with implementation details and documentation accessible at https://github.com/xleizi/easySCF.

## 1 Introduction

With the rapid advancement of single-cell RNA sequencing (scRNA-seq) technology and the significant reduction in sequencing costs, scRNA-seq has gradually become the preferred tool for studying cell types, developmental processes, disease mechanisms, and tumor heterogeneity. This technology allows researchers to reveal the heterogeneity of gene expression at the single-cell level, delving into the complexity and diversity of biological systems. scRNA-seq has been widely applied in the identification of cell types, analysis of developmental processes, research on disease mechanisms, and the analysis of tumor heterogeneity. However, the use of different data formats by various analysis tools presents certain technical challenges.

Currently, scRNA-seq analysis primarily relies on two major programming language platforms: R and Python. Popular analysis tools include R-based Seurat (Satija *et al.* 2015, Butler *et al.* 2018, Stuart *et al.* 2019, Hao *et al.* 2021, 2024), SCP (https://github.com/zhanghao-njmu/SCP), Monocle (Trapnell *et al.* 2014, Qiu *et al.* 2017a, 2017b, Cao *et al.* 2019), and Python-based Scanpy (Wolf *et al.* 2018), scVI (Gayoso *et al.* 2022, Virshup *et al.* 2023), and scbean (Zhang *et al.* 2024). Among these, Seurat and Scanpy are widely used due to their powerful functionalities. Despite this, issues with data format compatibility between different analysis platforms persist, increasing the complexity of data integration and analysis, and limiting the reproducibility and shareability of research findings. Seurat is extensively used in the field of bioinformatics for its excellent data normalization, dimensionality reduction, and clustering analysis capabilities, as well as its strong visualization features. Scanpy, on the other hand, is known for its efficient data processing capabilities and scalability, particularly suitable for handling large datasets. Its modular design and compatibility with the Python ecosystem also make it a popular choice in the field of single-cell analysis.

As researchers handle increasing volumes of single-cell data, many are analyzing millions of cells. With the release of the Visium HD spatial platform, a single HD dataset often contains up to 500,000 8 µm binned squares under tissue. Therefore, researchers frequently need to perform data analysis across different platforms. Commonly used data format conversion tools include anndata2ri (https://github.com/theislab/anndata2ri), scDIOR (Feng *et al.* 2022), SeuratDisk (https://github.com/mojaveazure/seurat-disk), Zellkonverter (https://github.com/theislab/zellkonverter), sceasy (Cakir *et al.* 2020) (https://github.com/cellgeni/sceasy), and MuDataSeurat (https://github.com/PMBio/MuDataSeurat). However, with the update of Seurat to version 5 (Hao *et al.* 2024), the spatial transcriptomics format transitioned from VisiumV1 to VisiumV2, rendering most of these conversion tools incompatible, and some of them complex to operate. The anndata2ri tool requires a SingleCellExperiment object to bridge the gap, enabling the conversion of Scanpy and Seurat objects into SingleCellExperiment objects for mutual transformation. Additionally, anndata2ri needs to call the R environment from Python, which may not be user-friendly for single-cell data analysis beginners. The scDIOR package had its last commits on 15 February 2022, and SeuratDisk’s last commits were on 4 November 2023. Zellkonverter is more inclined to facilitate data conversion between SingleCellExperiment and anndata. The sceasy package necessitates activating a Python environment via Conda from within R, which also poses a challenge for newcomers. Its last commits were on 2 September 2022. MuDataSeurat has successfully adapted to the Seurat version 5 format, but it does not support spatial transcriptomics data and struggles with data volumes exceeding 1 million cells, temporarily unable to handle large-scale sample conversions. Consequently, existing data format conversion packages are not well suited for practical data analysis processes in the face of the latest version of Seurat v5, large-scale data, and spatial transcriptomics data. To address this issue and better leverage the distinct data formats of Seurat and Scanpy, we developed a data format conversion tool that allows researchers to seamlessly switch data between Seurat and Scanpy. This tool not only enhances the flexibility of data analysis but also promotes data sharing and collaboration among different research teams. With this tool, researchers can fully exploit the strengths of both Seurat and Scanpy, thereby conducting more efficient analysis and interpretation of single-cell data.

This paper aims to introduce the design and implementation of a data conversion tool, detailing the mechanism of data format conversion between Seurat and Scanpy. We will demonstrate its potential in enhancing data analysis efficiency and result reproducibility through actual datasets. We hope that this tool will provide strong support for single-cell genomics research and further advance related studies.

When designing the single-cell data format conversion tool, we selected Hierarchical Data Format version 5 (HDF5) as the intermediary data storage format and stored the data as .h5 files. This choice was based on several key reasons and advantages: (i) Hierarchical data management: The HDF5 format allows the creation of complex hierarchical structures within a single file, organizing data into groups and subgroups. This is particularly suited for storing scRNA-seq data, facilitating efficient management and access. (ii) Efficiency in data storage: HDF5 is a highly efficient data storage format designed for handling large-scale datasets. Its structured data storage approach enables the storage of vast amounts of diverse datasets within the same file, while ensuring fast data reading and writing speeds. (iii) Cross-platform compatibility: The HDF5 format supports multiple programming languages and can be seamlessly read and written across different software environments. This greatly facilitates the conversion of data between Seurat and Scanpy. Researchers can preprocess and analyze data in the R environment using Seurat, then convert the data to HDF5 format for further analysis in the Python environment using Scanpy, and vice versa. Therefore, by choosing HDF5 as the intermediary storage format and storing data as .h5 files, we provide a solid foundation for the conversion of scRNA-seq data formats. This choice not only enhances the performance and reliability of our data conversion tool but also greatly facilitates data sharing and collaboration between researchers across different analysis platforms.

## 2 Methods

**Figure 1.**
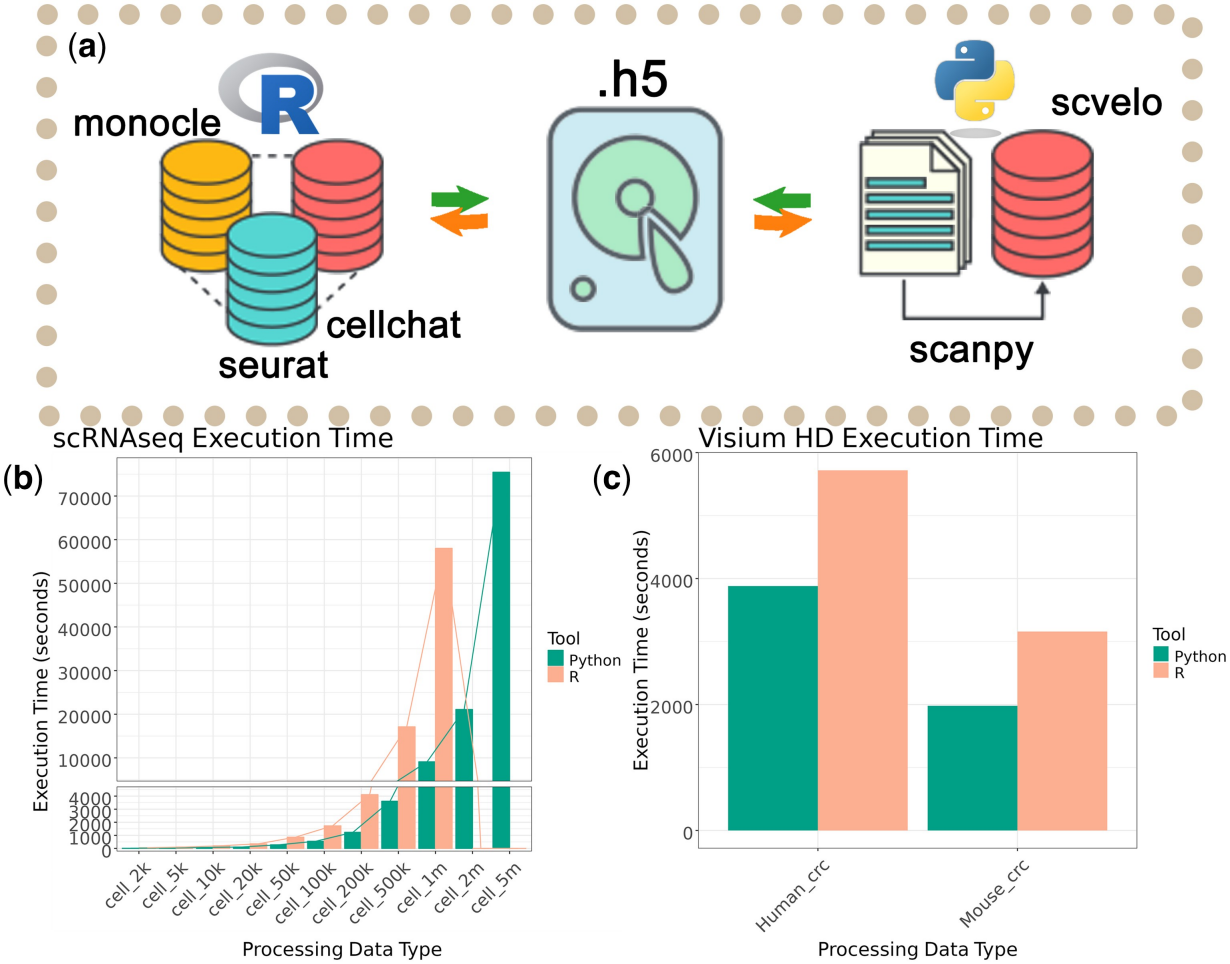
(a) Schematic diagram of data conversion. (b) Processing time test for scRNA. (c) Processing time test for Visium HD.

In order to facilitate data saving and loading between the R and Python platforms, we designed the easySCF package, which consists of two components: easySCTr for the R platform and easySCTpy for the Python platform. Both components ensure cross-platform compatibility by saving files in HDF5 format. In the Python environment, data reading and writing operations are primarily performed using the h5py and anndata libraries; in the R environment, we use the hdf5r package to accomplish similar tasks (Fig. 1a).

In the HDF5 file, we have created two datasets, names_obs and names_var, which are used to store cell names and gene names, respectively. Additionally, the file includes six groups: assay, obs, var, reductions, graphs, and images. Each group contains different types of data relevant to their respective categorizations. This organizational structure allows for efficient storage and easy access to a variety of biological and analytical data required for single-cell analysis.

**Figure 2.**
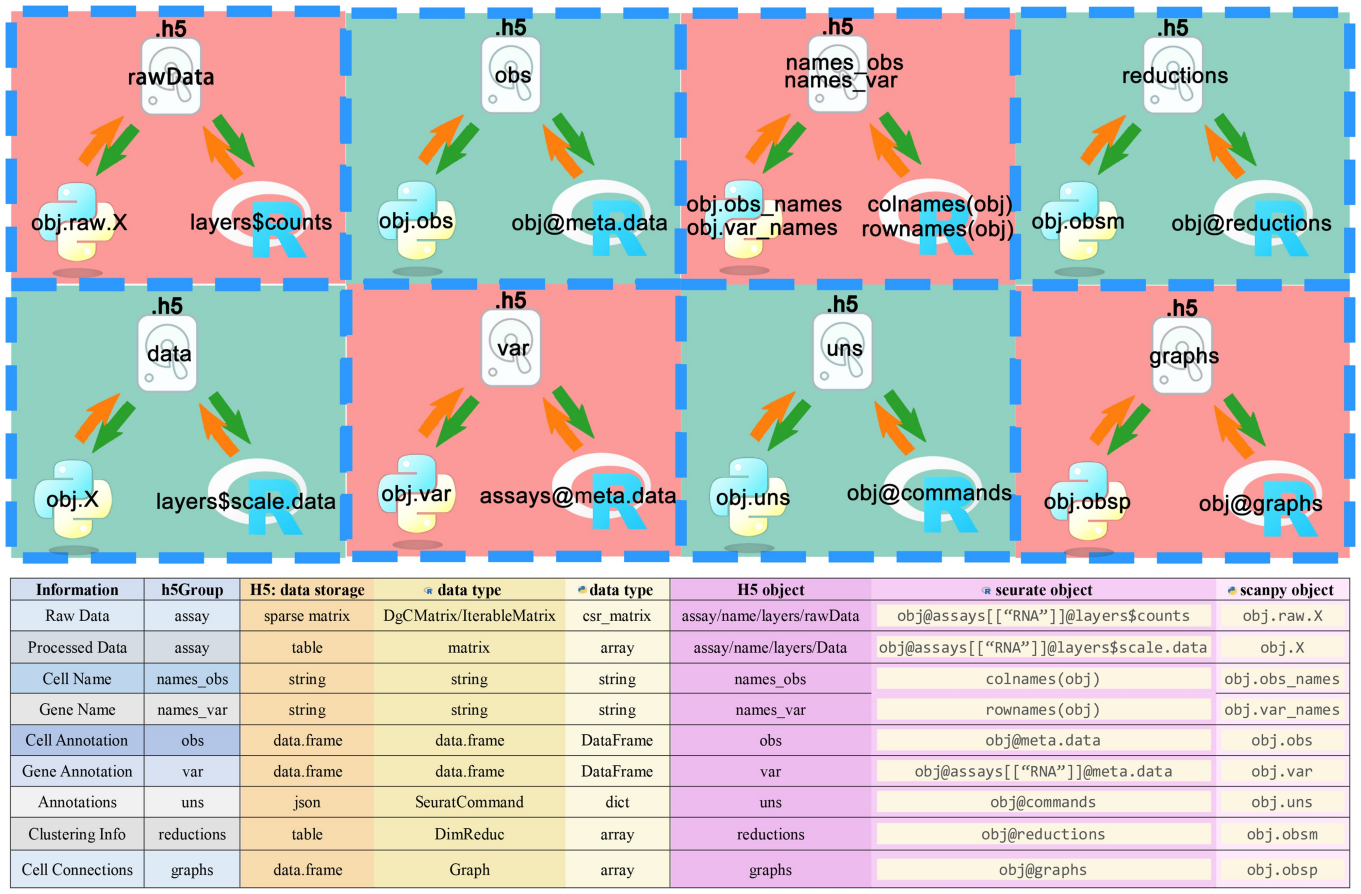
Schematic diagram of data storage structure.

In the assay group of the HDF5 file, the expression matrix for single cells is stored. In scRNA-seq, a sample contains many different types of cells. Each cell type expresses a specific set of genes and does not express most others. scRNA-seq has a detection limit and is susceptible to technical noise. As a result, even if some genes are expressed in the cell, they may not be detected due to low expression levels, appearing as zeros in the data. Therefore, in the raw counts data of single-cell analyses, only a small portion of genes show significant expression levels, while the expression of most genes is close to zero. To save storage space, the data in Scanpy are stored as compressed sparse row (CSR) matrices. We use the native write_elem and read_elem functions from the *anndata* library to handle reading and writing of the CSR matrix, which is structured into three dimensions: (i) data: An array that stores all nonzero elements. (ii) indices: An array that stores column indices corresponding to the nonzero elements. (iii) indptr: An array that stores the starting positions of nonzero elements in each row, with the data stored within the assay group. In Seurat, sparse matrices are stored in the dgCMatrix format (compressed, sparse, column-oriented numeric matrices), analogous to Python’s csc_matrix, a column-compressed sparse matrix format. When converting HDF5 data into Seurat matrices, we transpose the data to ensure consistency in the data format. Additionally, since the indptr values must be nondecreasing nonnegative integers, and in R, integers are int32 (ranging from −2^31 to 2^31 − 1), large single-cell datasets can exceed the int32 limit of indptr, causing data reading issues. To address this, we store every 5000 cells as one block, and for exceptionally large datasets, we store the expression matrix on disk using BPcells to manage large numbers of cells in a single layer in Seurat effectively (Fig. 2).

In both Seurat and Scanpy, the annotation files for cells and genes are stored in sce@meta.data and assay@meta.data, respectively, and in obs and var groups for the platforms. These tabular files are stored within the obs and var groups where each column data is stored in different datasets, and factor-type columns save each factor value in categories, while each row’s corresponding factor code is stored in codes to conserve storage space.

The reductions group is designated for storing dimensionality reduction data such as Principal Component Analysis (PCA), Harmony, Uniform Manifold Approximation and Projection (UMAP), and t-Distributed Stochastic Neighbor Embedding (t-SNE), corresponding to sce@reductions in Seurat and adata.obsm in Scanpy.

The graphs group holds data about the connections between cells, corresponding to sce@graphs in Seurat and adata.obsp in Scanpy. This includes *nn*, which is a nearest neighbor graph representing the relationships between each cell and its closest neighbors. These graphs are commonly used for cell clustering and classification. It also includes *snn*, or shared nearest neighbor graph, which builds upon the nearest neighbor graph by considering the number of shared neighbors between cells.

The images group supports spatial transcriptomics data type VisiumV2, better adapting to the latest Seurat format. In this group, we use a dataset named image to store color information for each pixel of the image, a dataset named coords to store the pixel position corresponding to each spot, and information about scale factors is stored in an HDF5 group named scale_factors. This arrangement not only provides a structured approach to storing complex spatial data but also facilitates the integration and analysis of spatial datasets in bioinformatics workflows.

## 3 Results

### 3.1 Performance comparison between Seurat and Scanpy

Our developed tool, easySCF (https://github.com/xleizi/easySCF), is designed to facilitate the conversion of single-cell data between R and Python, utilizing the .h5 format for unified data storage. easySCF comprises the “easySCFr” module in R and the “easySCFpy” module in Python. To assess the necessity of replacing Seurat with Scanpy when handling ultralarge datasets, we conducted sampling tests using data from the study titled “A single-cell transcriptional timelapse of mouse embryonic development, from gastrula to pup” (Qiu *et al.* 2023) available in the CELLxGENE database (cziscience.com) (Abdulla *et al.* 2023).

We sampled datasets containing 100, 200, 500, 1k, 2k, 5k, 10k, 20k, 50k, 100k, 200k, 500k, 1 million, 2 million, and 5 million cells, each with 45 854 genes. We then executed the standard workflow on both the R platform using Seurat and the Python platform using Scanpy, recording the data processing times.

Preliminary results indicate that the processing times using Scanpy were as follows: 12.65, 27.74, 75.16, 107.82, 278.76, 550.84, 1233.49, 3609.57, 9107.28, 21 090.35, and 75 452.26 s for datasets containing 100, 200, 500, 1k, 2k, 5k, 10k, 20k, 50k, 100k, 200k, 500k, and 1 million cells, respectively (Fig. 1b). In contrast, the processing times using Seurat were 53.23, 107.71, 203.60, 355.09, 861.04, 1726.77, 4110.90, 17 115.68, and 58 012.27 s for datasets up to 1 million cells, with datasets containing 2 million and 5 million cells being too large to process using the standard Seurat workflow, necessitating the use of SketchData for subsampling (Fig. 1c).

Our findings reveal that Scanpy processes data over three times faster than Seurat, with the speed advantage increasing as the dataset size grows. Additionally, we observed that when the dataset size is too large, with *P*-values exceeding 2^31, the standard Seurat workflow becomes unusable, requiring sketching methods for processing.

### 3.2 Efficiency in spatial transcriptomics analysis

In the field of spatial transcriptomics, we conducted analyses using the test data from the Visium HD datasets, specifically “Human Colorectal Cancer (FFPE)” (https://www.10xgenomics.com/datasets/visium-hd-cytassist-gene-expression-libraries-of-human-crc) and “Mouse Small Intestine (FFPE)” (https://www.10xgenomics.com/datasets/visium-hd-cytassist-gene-expression-libraries-of-mouse-intestine). When processing the human colorectal cancer data, Seurat required 5719.224 s, while processing the mouse small intestine data took 3158.841 s. In contrast, using Scanpy reduced the processing times to 3881.287 s and 1980.072 s, respectively, demonstrating a time savings of 32.13% and 37.31%. These results highlight the significant efficiency gains achieved by using Scanpy over Seurat and underscore the importance of adopting a multilanguage, multiplatform approach in data processing workflows.

We further extended our sampling tests on data from the CELLxGENE database, including additional dataset sizes at levels of 100, 200, 500, 1k, 2k, 5k, 10k, 20k, 50k, 100k, 200k, 500k, 1 million, 2 million, 5 million, and 10 million cells. These tests were conducted to evaluate read and write times, memory usage, and disk space utilization. By assessing these metrics across various dataset sizes, we aimed to gain a comprehensive understanding of the performance characteristics associated with different tools and platforms when handling large-scale single-cell data. This evaluation is crucial for optimizing workflows in terms of efficiency and resource management.

### 3.3 File reading and writing performance

**Figure 3.**
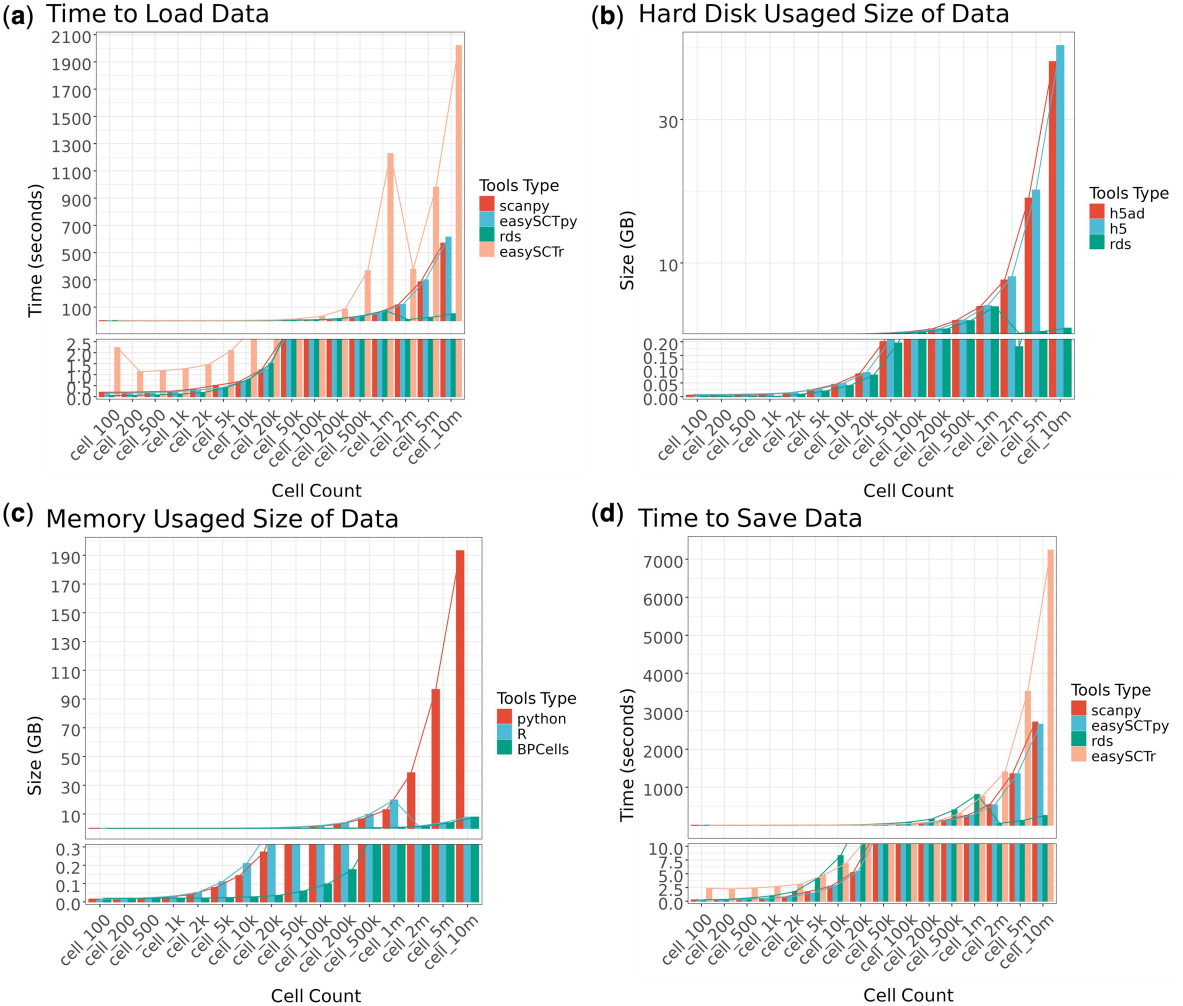
(a) Schematic diagram of data reading time. (b) Schematic diagram of data saving time. (c) Schematic diagram of memory usage. (d) Schematic diagram of disk space usage.

Regarding file reading performance, when using “readRDS” to read RDS files in R, the times were as follows: 0.05, 0.06, 0.08, 0.12, 0.19, 0.40, 0.78, 1.53, 3.76, 7.46, 14.88, 37.20, 74.73, 8.65, 24.84, and 50.86 s. In contrast, using “easySCFr” to read .h5 files took 2.23, 1.14, 1.19, 1.27, 1.46, 2.10, 3.14, 5.29, 15.08, 33.41, 84.90, 366.83, 1226.98, 377.12, 978.84, and 2019.95 s. In Python, reading .h5ad files using Scanpy took 0.20, 0.20, 0.22, 0.25, 0.34, 0.51, 0.67, 1.21, 2.74, 5.23, 10.28, 25.32, 50.42, 115.16, 285.10, and 569.12 s, while using “easySCFpy” took 0.16, 0.16, 0.19, 0.23, 0.30, 0.40, 0.69, 1.25, 2.96, 5.75, 11.35, 28.16, 56.10, 118.74, 300.06, and 613.97 s (Fig. 3a).

For file writing, using “saveRDS” in R to save files took 0.26, 0.33, 0.58, 0.98, 1.76, 4.20, 8.29, 16.29, 40.49, 80.82, 161.45, 404.50, 807.97, 50.72, 126.72, and 255.19 s, while using “easySCFr” to save .h5 files took 2.32, 2.23, 2.40, 2.68, 3.07, 4.72, 6.82, 11.74, 26.61, 50.72, 104.08, 306.54, 765.91, 1402.23, 3525.22, and 7240.29 s. In Python, the times for saving files using Scanpy and “easySCFpy” were 0.23, 0.25, 0.34, 0.51, 0.77, 1.71, 2.69, 5.20, 12.57, 25.94, 52.21, 129.16, 258.30, 540.59, 1353.29, 2715.47 s, and 0.23, 0.26, 0.35, 0.53, 0.79, 1.46, 2.82, 5.45, 13.40, 26.60, 53.05, 132.10, 264.95, 533.16, 1356.40, 2656.31 s, respectively (Fig. 3b).

These observations suggest that in Python, the read and write speeds of easySCF are comparable to those of native Scanpy, as easySCF utilizes Scanpy’s native “write_elem” and “read_elem” functions, both of which involve reading from HDF5 files. Therefore, the read and write speeds using easySCF are similar to those when using native Scanpy. However, in R, due to data format conversions, “readRDS” directly reads RDS-structured data in R, whereas easySCFr reads data from HDF5 structures and converts it to Seurat structures, resulting in longer read and write times compared to using “loadRDS” and “saveRDS”. Additionally, in R, when the dataset exceeds 1 million cells, easySCFr by default uses BPCells for data read and write operations. Leveraging the advantages of the BPCells data structure used in Seurat version 5, large-scale expression matrix data are processed directly on disk rather than being loaded into memory, which reduces read times. Therefore, during such operations, “loadRDS” and “saveRDS” do not perform disk-level read and write operations, making these methods relatively faster in handling large datasets.

### 3.4 Memory usage comparisons

We measured the memory usage after loading the sampled objects. In R, both readRDS and easySCFr read the same object, resulting in identical memory usage of 0.01, 0.02, 0.02, 0.03, 0.05, 0.11, 0.21, 0.40, 1.00, 1.97, 3.95, 9.85, 19.69, 1.58, 3.93, and 7.84 GB. When using the BPCells parameter, the memory usage was 0.02, 0.02, 0.02, 0.02, 0.02, 0.02, 0.03, 0.04, 0.06, 0.10, 0.18, 0.41, 0.80, 1.58, 3.93, and 7.84 GB.

In Python, the memory usage for objects loaded with Scanpy and easySCFpy was 0.01, 0.02, 0.02, 0.03, 0.04, 0.08, 0.14, 0.27, 0.66, 1.30, 2.60, 6.49, 12.97, 38.65, 96.59, and 193.14 GB. This indicates that, under standard storage methods, Python uses less memory than R. However, when dealing with extremely large datasets, using BPCells in R can significantly reduce memory consumption (Fig. 3c).

### 3.5 Disk space utilization

Regarding disk space, the sizes of the .h5ad files saved using Scanpy in Python were 0.0053, 0.0057, 0.0069, 0.0088, 0.0127, 0.0244, 0.0441, 0.0830, 0.2005, 0.3962, 0.7896, 1.9683, 3.9322, 7.6172, 19.0395, and 38.0679 GB. In R, the sizes of .rds files saved using saveRDS to save Seurat V5 object were 0.0016, 0.0019, 0.0031, 0.0008, 0.0088, 0.0205, 0.0401, 0.0787, 0.1953, 0.3885, 0.7780, 1.9433, 3.8858, 0.1818, 0.4524, and 0.9033 GB. The sizes of the .h5 files saved using easySCF were 0.0076, 0.0080, 0.0092, 0.0112, 0.0152, 0.0273, 0.0476, 0.0877, 0.2088, 0.4093, 0.8134, 2.0229, 4.0388, 8.0679, 20.1621, and 40.3121 GB. The data sizes across these different formats are similar. However, for datasets with 2 million, 5 million, and 10 million cells, the single-cell expression matrices are stored separately in the BPCells directory, leading to smaller .rds file sizes. These results demonstrate that easySCF is an efficient tool for reading and writing single-cell data, offering clear advantages in terms of memory and disk space usage. Compared to other data conversion software, easySCF can save more storage space and facilitate direct analysis of larger datasets, making it a highly effective tool for managing and analyzing single-cell data (Fig. 3d).

### 3.6 Optimized data conversion pathway

**Figure 4.**
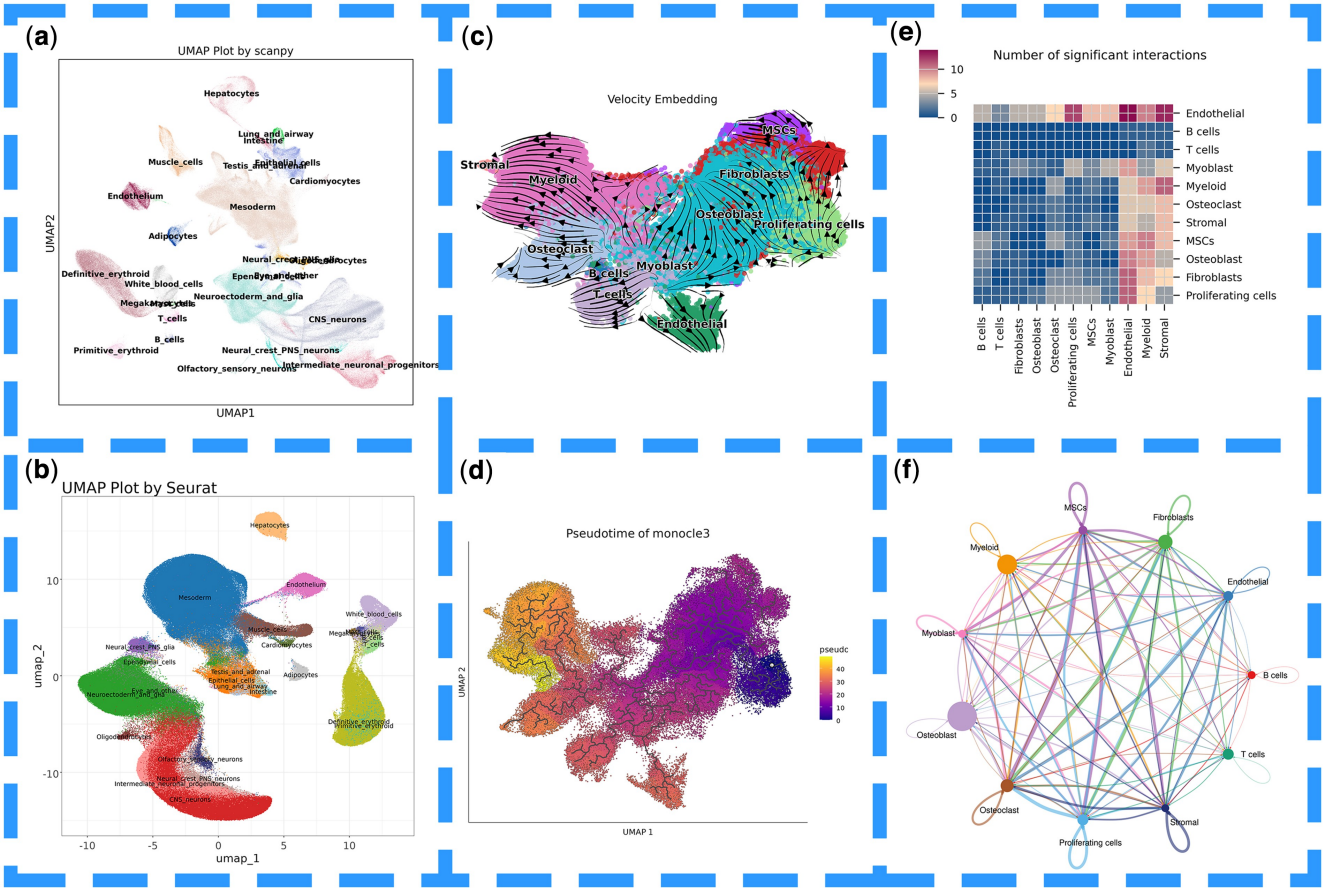
(a) Data processed using the standard Scanpy workflow. (b) Data processed using the standard Seurat workflow. (c) Data from GSE152048 converted from Seurat to Scanpy using easySCF and processed with scVelo. (d) Data from (c) converted from Scanpy to Seurat using easySCF and processed with Monocle3. (e) Data from (d) converted from Seurat to Scanpy using easySCF and processed with CellphoneDB. (f) Data from (e) converted from Seurat to Scanpy using easySCF and processed with CellChat.

Moreover, we have provided researchers with an optimized data conversion pathway (Fig. 4a and b), enabling them not only to use deep learning algorithms such as scVI in Python for dimensionality reduction and clustering but also to perform pseudotime analysis using Monocle3 (Cao *et al.* 2019) (Fig. 4d) and CellChat (Jin *et al.* 2021, Jin *et al.* 2024) (Fig. 4f) in R, or conduct cell factor receptor analysis with scVelo (Bergen *et al.* 2020) (Fig. 4c) and CellPhoneDB (Vento-Tormo *et al.* 2018, Efremova *et al.* 2020, Garcia-Alonso *et al.* 2021, Garcia-Alonso *et al.* 2022) (Fig. 4e) in Python. We tested this pathway using data from GSE152048 (Zhou *et al.* 2020), demonstrating its effectiveness in seamlessly converting data across different types of algorithms.

**Figure 5.**
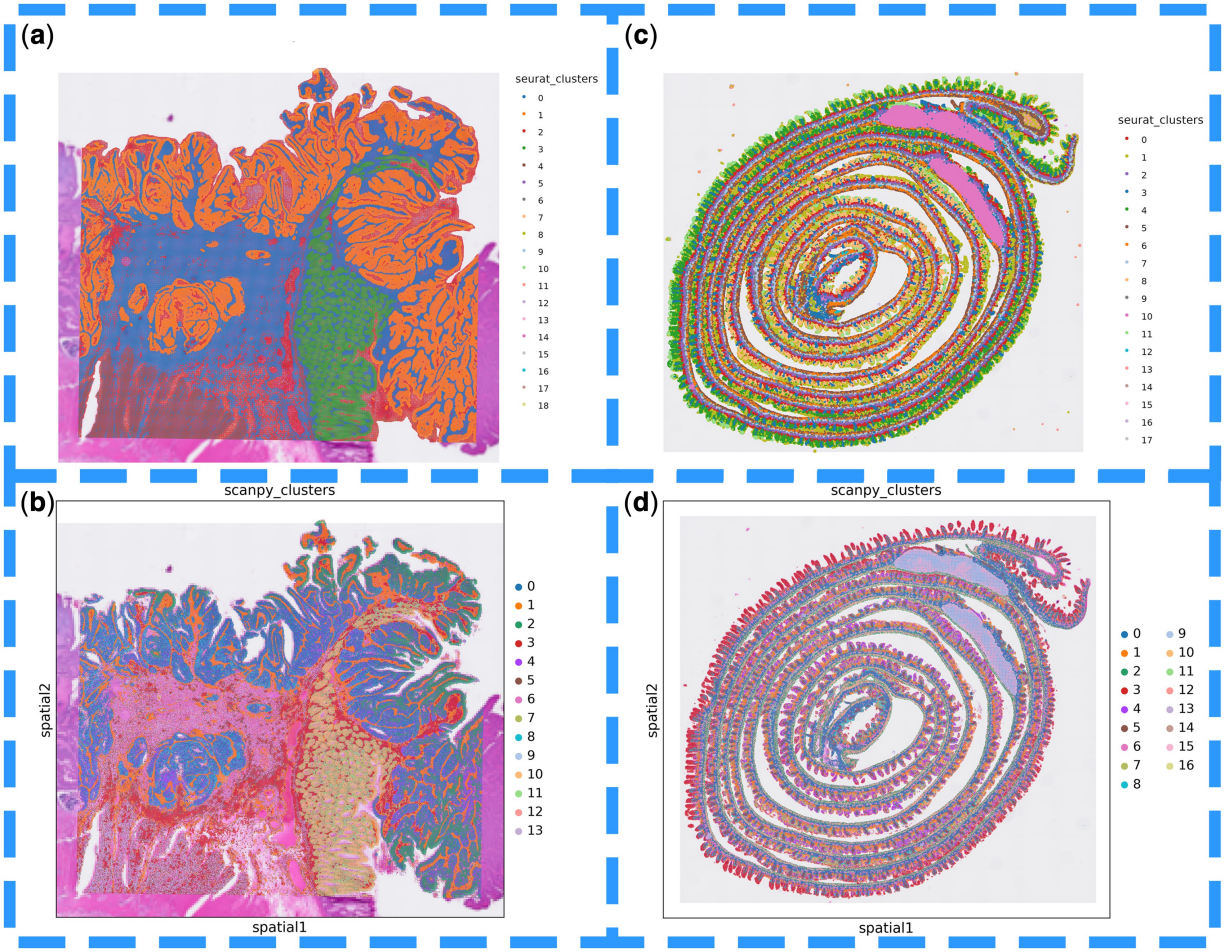
(a) easySCF is used to read the Visium_HD_Human_Colon_Cancer data saved by scanpy and processes it using the standard Seurat workflow. (b) easySCF is used to read the Visium_HD_Human_Colon_Cancer data saved by Seurat and processes it using the standard scanpy workflow. (c) easySCF is used to read the Visium_HD_Mouse_Small_Intestine data saved by scanpy and processes it using the standard Seurat workflow. (d) easySCF is used to read the Visium_HD_Mouse_Small_Intestine data saved by Seurat and processes it using the standard scanpy workflow.

Additionally, “easySCF” supports direct data conversion into the desired analysis format by adjusting the “readType” parameter, greatly simplifying the process for researchers to move directly into their analysis workflows. Figure 5a and b also illustrates the workflow for spatial transcriptomics processing in Seurat and Scanpy.

Overall, “easySCF” demonstrates exceptional performance in reading and writing single-cell data, offering significant storage space savings compared to traditional tools and improving user efficiency. Its simple and intuitive command structure also enhances ease of use and memorability for users. This is especially important in light of challenges faced by many tools in adapting to the structural changes introduced with the upgrade to Seurat V5, where frequent issues arise during data structure conversion. As a highly efficient and compatible tool with the latest versions, “easySCF” enhances the user experience, making it a robust solution in the field of single-cell data format conversion.

## 4 Conclusion

In this study, we successfully developed the easySCF tool to enhance the interoperability of single-cell data between R and Python. By standardizing data formats (.h5 format), easySCF greatly simplifies the process of data conversion between the two mainstream bioinformatics platforms. Our experimental results indicate that easySCF not only improves data processing speed but also significantly optimizes data storage and read/write efficiency. This tool facilitates more streamlined and effective analysis, enabling researchers to focus on advancing their scientific inquiries rather than struggling with data compatibility issues.

By comparing the performance of Seurat and Scanpy on the same dataset, we demonstrated that using easySCF in conjunction with Scanpy significantly enhances processing efficiency for large-scale single-cell datasets. This finding highlights the importance of adopting a multiplatform strategy in bioinformatics data analysis. Additionally, the performance of easySCF in terms of memory and disk usage efficiency further confirms its advantages in resource optimization. These results underscore easySCF’s role in facilitating more efficient and effective use of computational resources in the field of single-cell genomics.

The design of easySCF not only focuses on enhancing data processing efficiency and reducing resource consumption but also commits to supporting the latest data processing standards, such as version 5 of Seurat. This ensures the tool’s long-term viability and broad applicability among researchers. Additionally, easySCF’s user-friendly design makes it accessible and reduces the technical barriers that users often encounter in single-cell data analysis.

In summary, easySCF provides a powerful platform that supports researchers in efficiently converting and analyzing single-cell data between R and Python. We believe that the widespread adoption of this tool will contribute to advancing research in single-cell genomics, accelerating innovation and discovery in the biomedical field. Moving forward, we plan to continue expanding the functionalities of easySCF, supporting more data types and analytical tools to meet the growing demands of scientific research and further promote the development of the bioinformatics field.

Conflict of interest: The authors declare that the research was conducted in the absence of any commercial or financial relationships that could be construed as a potential conflict of interest.

## Data Availability

The source code for the easySCF package can be accessed at https://github.com/xleizi/easySCF. The data for read/write performance testing is derived from the dataset “A single-cell transcriptional timelapse of mouse embryonic development, from gastrula to pup,” available at https://cellxgene.cziscience.com/datasets. For the data conversion testing, the dataset was obtained from the GEO database under the accession number GSE152048.
